# Light Scattering by Commercial Sugar Solutions^1^

**DOI:** 10.6028/jres.063A.016

**Published:** 1959-12-01

**Authors:** Carl J. Rieger, Frank G. Carpenter

## Abstract

Using a direct measure of scattered light, it was found that commercial sugar solutions scatter light predominantly in a forward direction. The scattering at angles less than 30° was as much as one hundred times that at right angles to the incident beam.

It was found that the light scattering by commercial sugar solutions is inversely dependent on wavelength to a power of between 2 and 3, and that severe multiple scattering occurs when the turbidity of the solution is larger than 2×10^−1^cm^−1^ at 436 m*µ*. The scattering of commercial sugar solutions is compared with that of highly purified sucrose.

A method is discussed that will enable a good approximation of the turbidity of commercial sugar solutions to be made from a single forward scattering measurement at an angle of about 20° with respect to the incident light beam. A correction for scattered light in transmission measurements of these solutions is also introduced.

## 1. Introduction

Even though commercial sugar solutions have long been recognized as being somewhat turbid, there has never been a thorough study of their light-scattering behavior. Recently, however, light scattering by commercial sugar liquors has been receiving increased attention. It is a very sensitive measure of colloidal impurities, and the latter greatly influence the visual appearance of products such as beverages containing sugar.

Light scattering by highly purified sucrose solutions has been previously reported [1, 2, 3].^3^ It was found that the scattering of light by these solutions follows the Rayleigh law. Furthermore, the measured turbidity is in agreement with that calculated from osmotic pressure and molecular weight according to the relations derived by Debye [1].

Most of the previous work on the scattering of light by commercial sugar liquors has been done with transmission measurements [4, 5, 6, 7]. It was found that “turbidity” interfered with the determination of “color”, and, therefore, numerous methods were proposed to compensate for the effects of light scattering on transmission measurements. One of the earliest of these methods [5] consisted of making transmission measurements before and after a mechanical filtration. The portion of solution that was filtered was assumed to be free of “turbidity”, and the difference between the transmittancies was considered to be an estimate of “turbidity”. Another method employed transmission measurements in both the red and blue regions of the visible spectrum [6, 7]. The measurement in the red region was assumed to be affected only by scattering, while that in the blue was affected by both absorption and scattering. The difference (sometimes including an empirical factor) was interpreted as pure absorption in the blue. None of these methods proved entirely satisfactory, primarily because an unwarranted assumption was made or a simplification was attempted before the phenomenon was completely understood.

Direct measurements of scattered light, independent of any simultaneous absorption of light, can be made. Thus the correction for turbidity to be applied to transmission measurements can be evaluated directly. This direct measurement of scattered light has been used to conduct a systematic study of light scattering by commercial sugar solutions.

Only the optical factors involved in light scattering are considered in this paper. No attempt has been made to determine the chemical nature of the scattering particles. It is most probable that the scattering particles also absorb light and that the dissolved absorbing molecules also scatter light. However, the optical measurements discern only the overall scattering and absorption.

## 2. Terminology

To avoid confusion, the following terminology will be used. *Light scattering* will be the general term used to signify the broad aspects of the phenomenon, while *turbidity* will specifically refer to the amount of light scattered per unit path length as defined in either of the equivalent equations,

$$I=I_{0}e^{-\tau^{b}},\;\text{or}$$ (1)

−lnT=τb. (2)

In these equations *I* is the irradiance of transmitted light, *I*_0_ is the irradiance of incident light, *b* is the path length in cm, *T* is the internal transmittance, and *τ* is the turbidity in cm^−1^. These equations apply only to systems which scatter light with no absorption.

In systems that absorb light with no scattering, the Lambert-Beer law is applicable and can be written as follows:

$$\frac{-\log\;T}{bc}=a,$$ (3)

in which *c* is the concentration of the sugar in grams per milliter and *a* is the absorption index.

When the system both absorbs and scatters light, then one writes [8]

$$\frac{-\log\;T}{bc}=a*,$$ (4)

in which a* is the attenuation index.

Equation (2) for scattering with no absorption can be written in the same form as eq (3) and (4) and serves to define the scattering index, *s*, as follows:

$$\frac{-\log\;T}{bc}=\frac{\tau }{2.303c}=s.$$ (5)

For systems such as sugar solutions, which both absorb and scatter light, the attenuation is assumed to equal the sum of absorption and scattering. In terms of the attenuation, absorption, and scattering indices this can be written:

a*=a+s. (6)

In all of the above relations, the turbidity is expressed in terms of light lost from the transmitted beam. However, turbidity may also be evaluated by a direct measurement of all light scattered in all directions:

$$\tau =2\pi \int_{0}^{\pi }R_{\theta }\;\sin\theta \;d\theta ,$$ (7)

where θ is the angle of observation and *Re* is the Rayleigh ratio, expressed as:

$$R_{\theta }=\frac{i_{\theta }r^{2}}{I_{0}V},$$ (8)

where *r* is the distance between the scattering volume, *V*, and the observer, and *i*_θ_ is the intensity of scattered light.

The Rayleigh ratio is a fundamental parameter describing light scattering by any medium. It is essentially the ratio of scattered to incident light at a particular angle of observation. This is the quantity that is actually determined when scattering measurements are made.

In practice, the geometrical factors involved play a very important part, and a number of corrections must be made. These corrections have been adequately treated elsewhere [9, 10, 11] and need not be further discussed here.

## 3. Instrument Description

The instrument used to measure the scattered light was a slightly modified microphotometer.^4^ The light source was a mercury vapor lamp (GE, H100 A-4) with filters for isolating lines at wavelengths of 365, 436, and 546 m*µ*. The instrument was modified by decreasing the size of the slits in the incident and receiver optical systems in order to improve the angular resolution to about 1.5°. A cylindrical cell was used and the scattered light was detected by a high-sensitivity photomultiplier tube (1P21), which revolved about the cell from 0 to 145°, allowing angular scattering measurements to be made. The output of the phototube was amplified and recorded at a chart speed of 6 inches per minute. The recording greatly facilitated the “averaging out” of small fluctuations.

The instrument was calibrated with Ludox,^5^ according to the method proposed by Goring [12] and coworkers, to provide an absolute measure of turbidity.

## 4. Factors Influencing Light Scattering by Commercial Sugar Solutions

### 4.1. Angle of Observation

**Figure 1:**
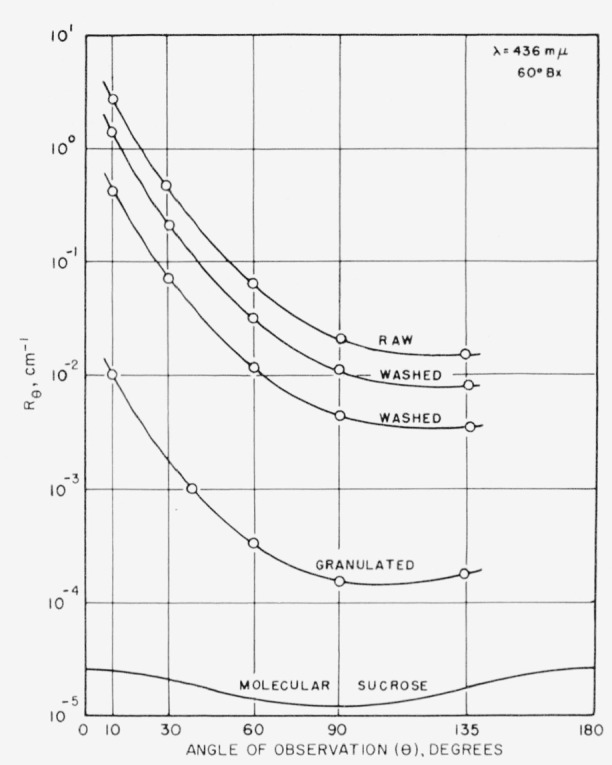
Angular scattering for some typical sugar solutions.

The dependence of light scattering on the angle of observation for some typical commercial sugar solutions is shown in figure 1. To remove large extraneous particles all solutions were filtered through coarse sintered glass. The concentration of the solutions was approximately 60 percent by weight of sugar solids, i.e., 60° Brix. Figure 1 indicates that these commercial sugar solutions scatter light predominately in a forward direction. Such behavior is usually interpreted [13] as resulting from a destructive interference of the light scattered from particles similar in size to the incident wave length.

Figure 1 also shows that the scattering of commercial sugar solutions is several orders of magnitude above the molecular scattering of sucrose.

**Figure 2:**
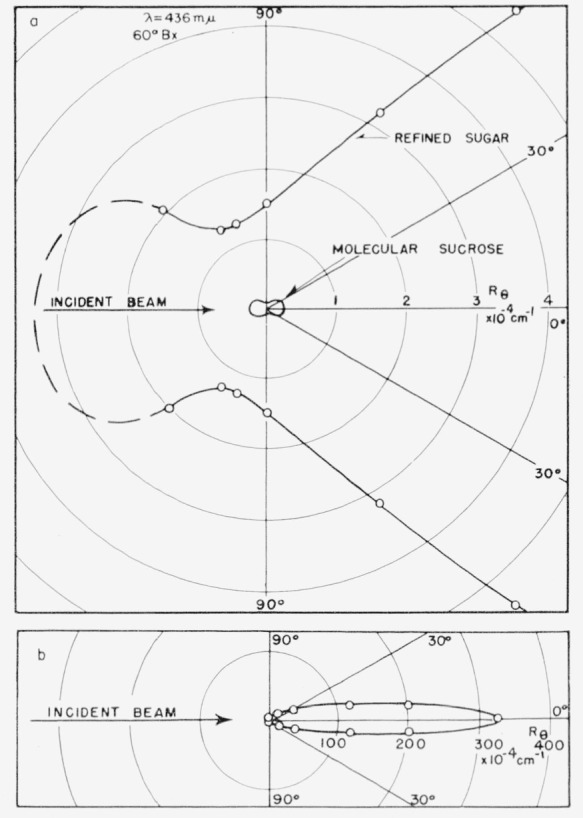
Scattering envelope of a refined sugar compared with that of molecular sucrose in polar coordinates. In figure 2b the scale has been decreased 100-fold to show the complete envelope of the refined sugar.

The significant difference between the scattering envelopes of purified sucrose solution and a typical commercial sugar can be seen more graphically when plotted in polar coordinates as in figure 2. The scattering envelope for pure sucrose is “peanut shaped”, typical of a Rayleigh-type scatterer, while the commercial sugar solution shows a predominantly forward-scattering envelope. To show the complete scattering envelope of a refined sugar, figure 2b is drawn on a decreased scale.

Scattering envelopes for a large number of commercial sugar solutions, covering a range of quality from impure raw sugars to the most highly purified sugar solutions, all have the shape of curve as in figures 1 and 2.

### 4.2. Refractive Index

To study the effects of refractive index on scattering by commercial liquors, a number of measurements on artificially controlled systems were made.

**Figure 3:**
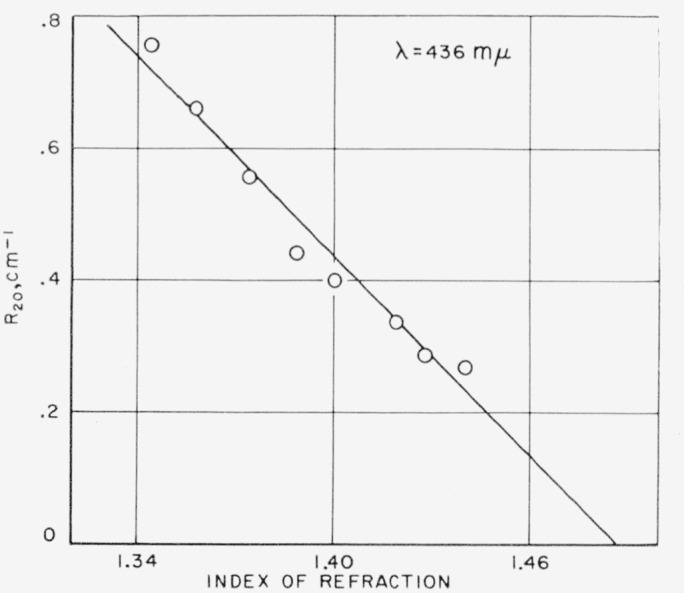
Effect of refractive index on scattering at constant concentration of scattering particles.

In the first experiment, the concentration of the scattering particles was held constant, and only the refractive index of the solution was changed. This was clone by adding a small constant amount of a raw sugar solution to varying proportions of highly purified sugar and water. The small scattering due to the highly purified sucrose-water mixture, considered as the solvent, was subtracted from the total scattering to obtain that due to raw sugar alone. Figure 3 shows that scattering decreased as the refractive index increased, when the concentration of scattering material was held constant. This is explained by the well known fact that light scattering results from a difference in refractive index between the medium and suspended particles. If the scattering particles have a refractive index above that of the solution, then as the refractive index of the solution is raised, the difference becomes less and there is less scattering. A linear extrapolation of the data in figure 3 approaches zero scattering at a refractive index of about 1.49, which can be interpreted as an “average” index of refraction of the particles causing the light scattering.

**Figure 4:**
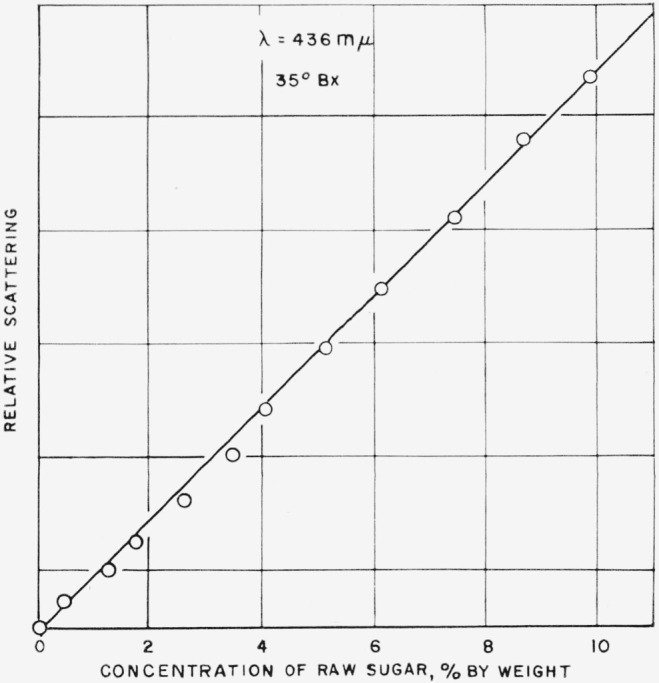
Effect of concentration of scattering particles at constant refractive index.

Figure 4 shows the opposite case, where the refractive index of the solution is held constant, and only the concentration of the particles is changed. This was accomplished by adding various small known amounts of a raw sugar to a highly purified sucrose solution. It can be seen from figure 4 that the scattering at constant refractive index is directly proportional to concentration in this system.

**Figure 5:**
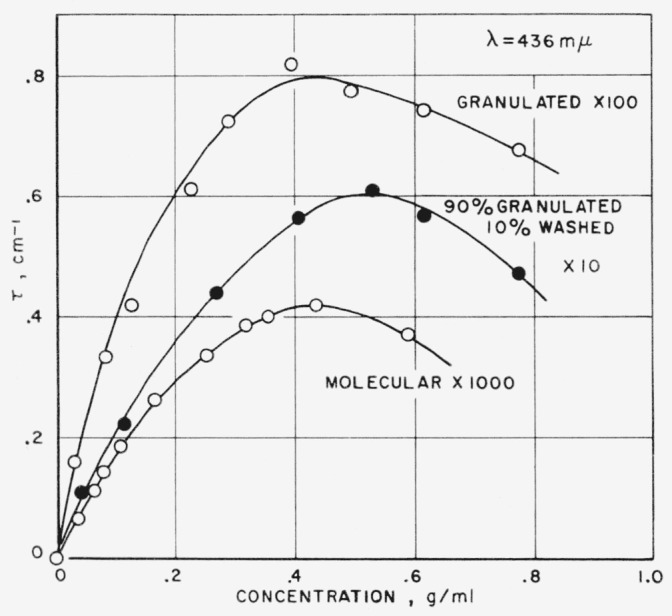
Effect of sucrose concentration on turbidity.

In figure 5 is shown a plot of turbidity as a function of sucrose concentration for the more usual case in which sugar is diluted with water, where both refractive index and sucrose concentration change. Even though the sugars are of different levels of turbidity, it is interesting that all show a maximum of turbidity at about a sucrose concentration of 0.4 g/ml (i.e., ~35° Brix). This decrease in turbidity above a certain concentration has been previously explained for solutions of highly purified sucrose by Halwer [2]. It may also be explained (for commercial sugars) as the combination of the effects shown in figure 3 and figure 4, that is, the turbidity increases with concentration up to a point, then as the refractive index of the medium begins to approach that of the particles, the turbidity decreases.

### 4.3. Wavelength Dependence

When the linear dimensions of the scattering particles are less than about 120 the wavelength of incident light, the total amount of light scattered is inversely proportional to the fourth power of the wavelength (Rayleigh scattering). For larger particles, the wavelength exponent will be less than 4, approaching 2 for particles comparable in size to the wavelength of light, and is zero for very large particles [14].

The dependence of scattering on wavelength for some commercial sugar solutions was determined at wavelengths of 365, 436, and 546 m*µ*. A log-log plot of turbidity as a function of wavelength resulted in straight lines the slopes of which were the wavelength exponents. The wavelength exponent for commercial sugar solutions varied between 2 and 3, corresponding to a particle size comparable to the wavelength, in agreement with the particle size deduced from the shape of the scattering envelope.

### 4.4. Multiple Scattering

Two of the major difficulties encountered in the measurement of light scattered by raw sugar solutions are multiple scattering and high absorption.

Multiple scattering occurs when the solution is so turbid that the light scattered by one particle is rescattered before it leaves the cell. The result is an abnormal increase in scattered light at wide angles. There is no simple quantitative interpretation for multiple scattering, and the turbidities measured in the usual way for such systems are only “apparent” turbidities.

**Figure 6:**
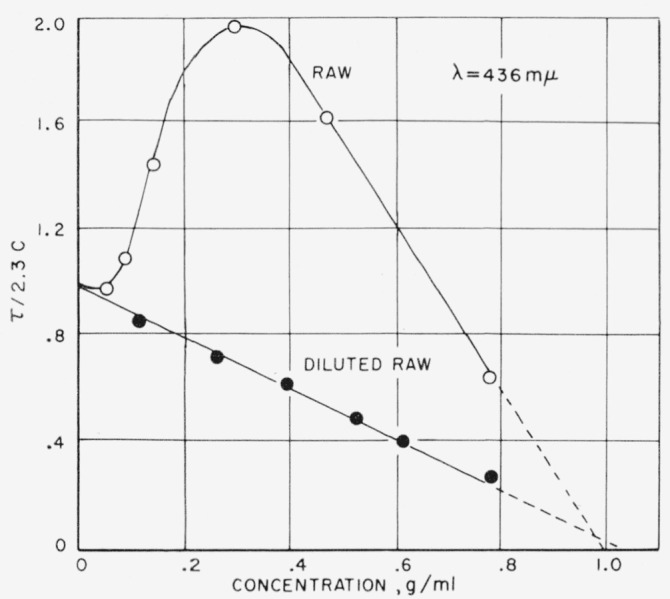
Effect of multiple scattering. The lower curve represents a 10-fold dilution of the raw sugar, plotted (10x) to obtain a comparison.

The effect of multiple scattering is shown vividly in figure 6, where the scattering index is plotted as a function of sucrose concentration for a raw sugar solution and for the same solution diluted 10-fold with a purified sugar solution of the same refractive index. Figure 6 shows that in the dilute concentration range both solutions have approximately the same scattering index. However, as concentration is increased, the multiple scattering of the raw sugar solution becomes much greater. At still higher concentrations both curves tend toward zero turbidity because the difference in refractive indexes between particles and solution tends toward zero. Because of multiple scattering, the values obtained for the undiluted solution cannot be quantitatively interpreted.

It is at once evident that to make valid scattering measurements on raw sugar solutions, it is necessary to dilute these solutions to a concentration range where multiple scattering does not occur. After a correction for the solvent scattering (purified sugar solution), it is possible to extrapolate to the original raw sugar concentration and obtain a value for the scattering as if multiple scattering did not occur. It was found that most commercial sugar solutions do not show severe multiple scattering until turbidity values larger than about 0.2 cm^−1^ at 436 m*µ* are reached.

### 4.5. Color

Light scattered by highly colored solutions can be evaluated by applying a suitable correction for the light lost by absorption. It has been shown [15] that if the cell is centered, and if the transmitted irradiance is measured instead of the incident irradiance, the correction is automatically applied, because both the transmitted and scattered light beams will be attenuated through the same cell path. The presence of a very dark color does complicate scattering measurements by absorbing so much light that little response is obtained from the phototube.

The dilution method mentioned above therefore serves a two-fold purpose, it eliminates multiple scattering and reduces the amount of absorption present, thus enabling valid scattering measurements to be made on raw sugar solutions.

## 5. Approximation of Sugar Turbidity From a Single Scattering Measurement

**Figure 7:**
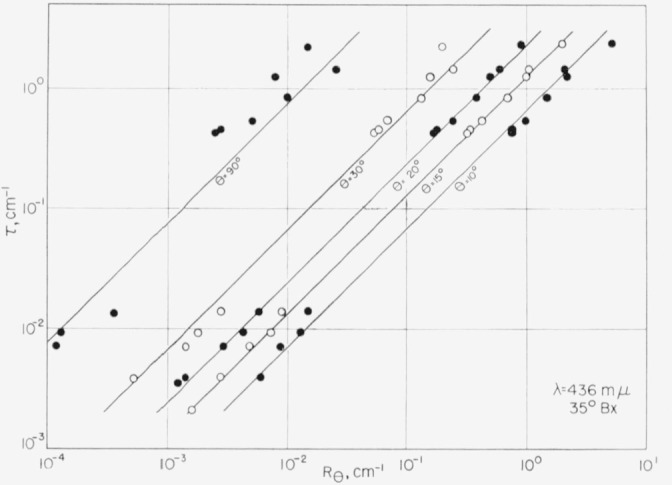
Correlation between the turbidity and R_θ_ at various angles of observation.

Turbidity, which is the total scattering integrated over all angles, is evaluated by integrating eq (7). However, when the shape of the scattering envelope is always the same, a simplification is possible because the entire envelope can be defined by a measurement at any one point. This is commonly done in the case of Rayleigh scatters [16] to obtain the relation between *τ* and *R*_90_. As was previously noted in figure 1, commercial sugars also have scattering envelopes of nearly the same shape (although not the same as a Rayleigh scatterer). Therefore, their turbidity can also be estimated from a single measurement. To determine the particular angle that would give the best evaluation of turbidity, the turbidities of a large number of sugars were determined by graphically integrating eq (7). These turbidities are shown in figure 7 as a function of *R_θ_* at various angles of observation.

The best correlation between turbidity and *R*_θ_ is obtained when the angle of observation, θ, is approximately 20°. The very poor correlation obtained when θ is 90° shows that right angle scattering measurements are of little value in determining turbidity of commercial sugar liquors.

**Figure 8:**
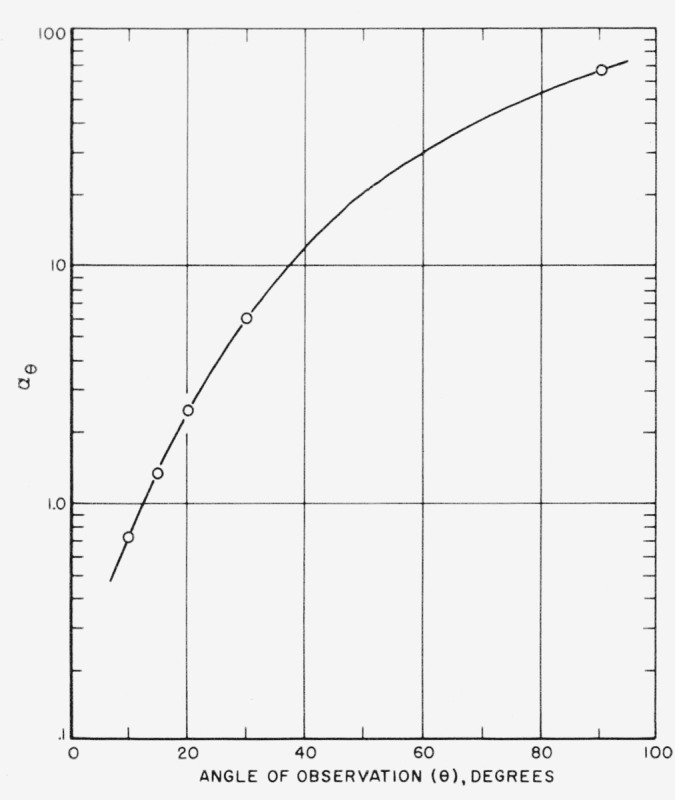
Dependence of α_θ_ on angle of observation.

Since figure 7 is a log-log plot and the slope of all the lines is 1.00, the general equation is of the form:

$$\tau =\alpha_{\theta }R_{\theta },$$

where *α*_θ_ is the intercept at *R*_θ_ = 1. The value of *α*_θ_ depends on θ as shown in figure 8. This constant is not dependent upon the instrument used and is applicable to most commercial sugars and to several other similar turbid materials having scattering envelopes of the same shape. It is noted that *α*_90_ is about 70, which can be compared with 16.75 for a Rayleigh scatterer. The choice of 20° for the angle of observation is subject to some latitude. However, the actual angle must be precisely known so that the appropriate value of *α*_θ_ can be chosen from figure 8. The value at 20° is 2.45.

## 6. Separation of Absorption and Scattering by Optical Means

It is evident from eq (6) that the absorption index is the difference between the attenuation index and scattering index. The attenuation can be measured with a spectrophotometer^6^ and the turbidity can be independently determined from scattering measurements or approximated from a single measurement as discussed above. A method is thus provided for correcting attenuation for scattering to obtain the true absorption.

**Figure 9:**
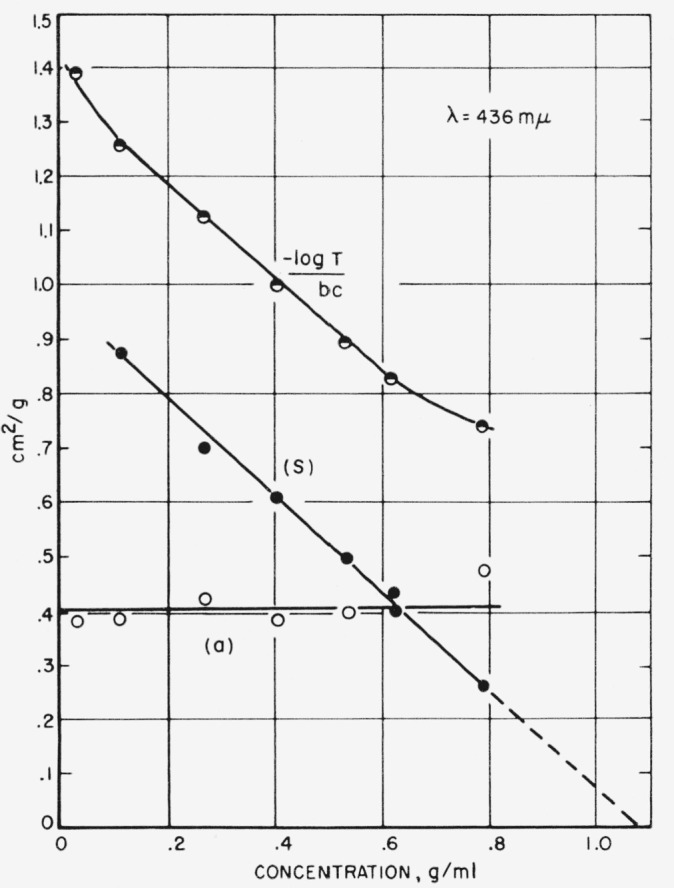
Effect of concentration on attenuation, absorption, and scattering indices for a typical washed sugar.

Figure 9 and table 1 show the results of a separation of absorption and scattering for some typical commercial sugar liquors. The attenuation index, the absorption index, and the scattering index are plotted in figure 9 as a function of concentration of sugar solids. The attenuation index and scattering index are not independent of concentration, but decrease with increasing concentration, because of the refractive index effects discussed in section 4.2. This stresses the need to specify the concentration when reporting values for these quantities.

Table 1 shows that even in granulated sugar liquors an appreciable percentage of the light lost is due to scattering. Both the scattering index and the absorption index are reduced by a factor of 10 to 100 by the refining process.

## 7. Conclusions

To characterize the overall scattering of commercial sugar liquors completely, it is necessary to make angular scattering measurements. Since the scattering envelopes of most commercial sugar solutions have approximately the same shape, a good approximation of the total turbidity may be made from a single scattering measurement.

A method is proposed that will enable a separation of absorption and scattering to be made from a single transmission and a single scattering measurement.

## Figures and Tables

**Table 1:** Separation of absorption and scattering of sucrose solutions λ=436 m*µ*; concentration = ~35° Brix

Type	a^*^ $\left(\frac{-\log\;T}{bc}\right)$	*s* $\left(\frac{\tau }{2.303c}\right)$	*a* (a*–s)	Percent light lost by scattering
				
Granulated:				
Medium	0.0315	0.0238	0.0077	75.5
Medium	.0134	.0066	.0068	49.3
Fine	.0722	.0391	.0331	54.2
Tablets	.0452	.0182	.0270	40.3
Washed	.7072	.428	.2792	60.5
Washed	.6154	.400	.2154	65.0
Soft	22.66	2.16	20.5	9.53
Hawaiian raw	4.04	0.507	3.53	12.5
Cuban raw	5.56	.782	4.78	14.1

## Footnotes

3 Figures in brackets indicate the literature references at the end of this paper.

4 Manufactured by the American Instrument Co., Silver Spring, Md. Described in detail by G. Oster, *Anal. Chem*. 25, 1176 (1953).

5 Colloidal silica, manufactured by E. I. du Pont de Nemours Co., Inc., Grasselli Chemical Dept., Wilmington, Del.

6 Since most commercial sugars scatter light predominately forward, precaution must be taken to eliminate most of the scattered light from reaching the phototube. This can be accomplished by using a very small slit or pin hole in front of the phototube.
